# Eating behaviour among adults with different levels of emotional suppression and eating disorder symptomatology

**DOI:** 10.1192/j.eurpsy.2022.402

**Published:** 2022-09-01

**Authors:** A. Brytek-Matera, K. Czepczor-Bernat, P. Bronowicka, A. Modrzejewska, J. Modrzejewska, P. Szymańska, J. Waliłko

**Affiliations:** 1 SWPS University of Social Sciences and Humanities, Faculty Of Psychology In Katowice, Katowice, Poland; 2 University of Wroclaw, Eat Lab (eating Behavior Laboratory), Wroclaw, Poland

**Keywords:** adults, eating disorder risk, eating behaviour, emotion regulation

## Abstract

**Introduction:**

Research has shown that emotional suppression, a form of emotion regulation, is often used by individuals with disordered eating behaviour. Moreover, eating disorder symptomatology is associated with inappropriate eating behaviours (e.g. excessive consumption of high-calorie foods and comfort foods).

**Objectives:**

The objective of the present study was to investigate the differences in eating behaviour among adults with different levels of emotional suppression and eating disorder symptomatology.

**Methods:**

Two hundred seventy adults (*M*_age_ = 29.44 ± 9.32) completed the Three-Factor Eating Questionnaire (eating behaviour), the Eating Attitudes Test (eating disorder symptomatology) and the Emotion Regulation Questionnaire (emotional suppression).

**Results:**

Three clusters were identified through cluster analysis: cluster 1 (*N* = 115) presenting low emotional suppression and low eating disorder symptomatology; cluster 2 (*N* = 43) presenting high emotional suppression and high eating disorder symptomatology and cluster 3 (*N* = 112) presenting high emotional suppression and low eating disorder symptomatology. Our results showed that individuals in cluster 2 had significantly greater levels of cognitive restraint, uncontrolled eating and emotional eating than individuals in clusters 1 and 3. Moreover, individuals in clusters 1 and 3 did not differ significantly in terms of any of the TFEQ subscales.

**Conclusions:**

These preliminary findings may suggest that the tendency to persistently suppress emotions exacerbate disordered eating behaviour. Therefore, this factor together with symptoms of eating disorders should to be considered when planning prevention and intervention programs among adults presenting disordered eating behaviour.

**Disclosure:**

No significant relationships.

